# Subjective benign paroxysmal positional vertigo in patients with osteoporosis or migraine^☆^

**DOI:** 10.1016/j.bjorl.2018.10.003

**Published:** 2018-11-06

**Authors:** Rocío González-Aguado, Esther Domènech-Vadillo, María Guadalupe Álvarez-Morujo de Sande, Gloria Guerra-Jiménez, Emilio Domínguez-Durán

**Affiliations:** a Hospital Universitario Marqués de Valdecilla, Santander, Spain; b Hospital Universitario Joan XXIII, Tarragona, Spain; c Hospital Universitario Puerta del Mar, Cádiz, Spain; d Complejo Hospitalario Universitario Insular Materno-Infantil, Las Palmas de Gran Canaria, Spain; e Hospital Infanta Luisa, Sevilla, Spain

**Keywords:** BPPV, Migraine disorders, Osteoporosis, VPPB, Distúrbios da migrânea, Osteoporose

## Abstract

**Introduction:**

Subjective benign paroxysmal positional vertigo is a form of benign paroxysmal positional vertigo in which during the diagnostic positional maneuvers patients only present vertigo symptoms with no nystagmus.

**Objective:**

To study the characteristics of subjects with subjective benign paroxysmal positional vertigo.

**Methods:**

Prospective multicenter case-control study. All patients presenting with vertigo in the Dix-Hallpike test that presented to the participating hospitals were included. The patients were separated into two groups depending on whether nystagmus was present or not. An Epley Maneuver of the affected side was performed. In the follow-up visit, patients were checked to see if nystagmus and vertigo were present. Both groups of patients were compared to assess the success rate of the Epley maneuver and also to compare the presence of 19 variables.

**Results:**

259 patients were recruited, of which 64 belonged to the subjective group. Nystagmus was eliminated in 67.2% of the patients with benign paroxysmal positional vertigo. 89.1% of the patients with subjective benign paroxysmal positional vertigo remained unaffected by nystagmus, thus showing a significant difference (*p* = 0.001). Osteoporosis and migraine were the variables which reached the closest to the significance level. In those patients who were taking vestibular suppressors, the percentage of subjective benign paroxysmal positional vertigo was not significantly higher.

**Conclusions:**

Subjective benign paroxysmal positional vertigo should be treated using the Epley maneuver. More studies are needed to establish a relationship between osteoporosis, migraine and subjective benign paroxysmal positional vertigo. The use of vestibular suppressants does not affect the detection of nystagmus.

## Resumo

**Introdução:**

A vertigem posicional paroxística benigna subjetiva é um tipo de vertigem posicional paroxística benigna na qual, durante as manobras posicionais diagnósticas, os pacientes apresentam apenas sintomas vertiginosos sem nistagmo.

**Objetivo:**

Estudar as características de indivíduos com vertigem posicional paroxística benigna subjetiva.

**Método:**

Estudo prospectivo multicêntrico de caso-controle. Foram incluídos todos os pacientes com vertigem no teste de Dix-Hallpike, que se apresentaram nos hospitais participantes. Os pacientes foram separados em dois grupos, dependeu da presença ou não do nistagmo. Uma manobra de Epley foi realizada no lado afetado. Na consulta de seguimento, os pacientes foram avaliados para verificar a presença ou não do nistagmo e da vertigem. Ambos os grupos de pacientes foram comparados para avaliar a taxa de sucesso da manobra de Epley e também para comparar a presença de 19 variáveis.

**Resultados:**

Foram recrutados 259 pacientes, dos quais 64 pertenciam ao grupo subjetivo. O nistagmo foi eliminado em 67,2% dos pacientes com vertigem posicional paroxística benigna. Em 89,1% dos casos, os pacientes com vertigem posicional paroxística benigna subjetiva mantiveram-se não afetados pelo nistagmo, mostraram uma diferença significativa (*p* = 0,001). Osteoporose e enxaqueca foram as variáveis que atingiram o nível mais próximo ao de significância. Nos pacientes que tomavam supressores vestibulares, a porcentagem de vertigem posicional paroxística benigna subjetiva não foi significativamente maior.

**Conclusões:**

A vertigem posicional paroxística benigna subjetiva deve ser tratada com a manobra de Epley. Mais estudos são necessários para estabelecer uma relação entre osteoporose, enxaqueca e vertigem posicional paroxística benigna subjetiva. O uso de supressores vestibulares não afeta a detecção do nistagmo.

## Introduction

Benign paroxysmal positional vertigo (BPPV) is caused by otoconia That are dislodged from the otolith macula beds and are trapped in a semicircular canal. There, gravity causes them to move after changes of the head position in the plane of the affected canal.^1^ BPPV is probably the most common cause of vertigo, with a lifetime prevalence of 2.4%.^2^ BPPV is diagnosed using the Dix-Hallpike^3^ or Pagnini-McClure^4^, ^5^ maneuvers.

In 2015, the Bárány Society produced a consensus document to help doctors around the world to diagnose BPPV. This document standardizes the diagnosis of BPPV using certain diagnostic criteria. According to these criteria, the definite diagnosis of BPPV requires diagnostic positional maneuvers that lead to the observation of a canal-specific positional nystagmus.^1^ However, in clinical practice it is not unusual to meet patients who present symptoms that strongly suggest BPPV, but then during the diagnostic positional maneuvers only experience vertigo symptoms with no nystagmus. This situation was described in 1994 by Weider et al.^6^ In 2002, Haynes et al. coined the term “subjective BPPV” (sBPPV) to describe this phenomenon.^7^ Patients with sBPPV complain of vertigo suggestive of BPPV, but do not have nystagmus observable to the examiner during Dix-Hallpike or Pagnini-McClure testing. The Bárány Society includes sBPPV in the category of “possible BPPV”.

The absence of nystagmus in sBPPV has been attributed both to fatigability of nystagmus after repeated testing as well as to a less noxious form of BPPV in which the neural signal is strong enough to elicit vertigo, but not strong enough to reach the threshold necessary to stimulate the vestibulo-ocular pathway.^7^ The side on which the symptoms are triggered in sBPPV should be treated since the symptoms remission rate is 67.64%.^8^ This finding suggests that sBPPV shares physiopathological mechanisms with BPPV. However, sBPPV is a less well-known phenomenon because in general these patients, who do not have nystagmus, are taken off the studies,^8^ which is probably because it is difficult to rule out alternative diagnoses for them.

The objective of this study is to study the characteristics of subjects with sBPPV compared with subjects who meet the criteria established by the Bárány Society for BPPV of the posterior semicircular canal.

## Materials and methods

All patients with BPPV that presented to the Otoneurology Units of the five participating hospitals were prospectively recruited between April 1st, 2015 and March 31st, 2016. Those Patients who presented spontaneous nystagmus or BPPV of the horizontal semicircular canal were excluded from this study.

All patients underwent left and right Dix-Hallpike Maneuver (DHM)^3^ once, without Frenzel glasses. Patients who did not demonstrate nystagmus or vertigo in any of the DHM were excluded. Patients presenting nystagmus both in the right and left DHM, and patients in whom any of the maneuvers triggered nystagmus different from canalolithiasis of the posterior canal were also excluded.

After this, the patients were separated into two groups. The first group, objective BPPV (oBPPV), was composed of patients who presented a nystagmus compatible with canalithiasic involvement of only one of the posterior semicircular canals. The second group was made up of patients with sBPPV, i.e. those who reported vertigo symptoms in one of the DHM, but did not present nystagmus. Data on all the variables defined by Table 1 was collected for all of the patients who met the criteria for inclusion in the study.

**Table 1:** Variables collected from the patients who joined the study. A description for each variable is shown, as well as the different categories for each of them, the number of subjects in the sBPPV and oBPPV groups and the *p*-value of the *χ*^2^ test or the Mann–Whitney *U* test.

Variable	Description	Categories	sBPPV	oBPPV	*p*-value
*Demographic variables*					
Sex	Man or woman	Man	25.0% (16)	28.7% (56)	0.565
		Woman	75.0% (48)	71.3% (139)	
Age	Age in years	0–49	32.8% (21)	28.7% (56)	0.534
		>50	67.2% (43)	71.3% (139)	

*Cardiovascular risk factors*					
Hypertension	Diagnosis after periodic screening studies in primary healthcare	No	57.8% (37)	63.4% (123)	0.424
		Yes	42.2% (27)	36.6% (71)	
Diabetes	Diagnosis after periodic screening studies in primary healthcare	No	92.2% (59)	93.3% (181)	0.762
		Yes	7.8% (5)	6.7% (13)	
Hyperlipidemia	Diagnosis after periodic screening studies in primary healthcare	No	75.0% (48)	72.7% (141)	0.716
		Yes	25.0% (16)	27.3% (53)	
Smoking habit	Active or non-active smoker	Non-active	82.8% (53)	82.5% (160)	0.951
		Active	17.2% (11)	17.5% (34)	
Alcohol consumption	Any alcohol intake within 24-h period prior to medical consultation	No	79.7% (51)	78.4% (152)	0.821
		Yes	20.3% (13)	21.6% (42)	

*Factors related to development of BPPV*					
Migraine	Past or current history of migraine	No	48.4% (31)	62.1% (121)	0.055
		Yes	51.6% (33)	37.9% (74)	
Osteoporosis	Osteoporosis diagnosed through densitometry within the previous 2 years	No	14.3% (3)	43.1% (22)	0.064
		Osteopenia	42.9% (9)	25.5% (13)	
		Osteoporosis	42.9% (9)	31.4% (16)	
Traumatic brain injury	Traumatic brain injury within a maximum of 90 days before the start of the current vertigo attack	No	95.3% (61)	91.8% (179)	0.349
		Yes	4.7% (3)	8.2% (16)	
Traffic accident	Sudden head deceleration or whiplash-like cervical injury within a maximum of 90 days before the start of the current vertigo attack	No	95.3% (61)	96.4% (188)	0.692
		Yes	4.7% (3)	3.6% (7)	
Prolonged head extension	Prolonged head extension the day before the start of the current vertigo attack	No	92.2% (59)	84.6% (165)	0.124
		Yes	7.8% (5)	15.4% (30)	

*BPPV characteristics*					
First episode	First episode of BPPV	No	68.3% (43)	59.8% (113)	0.231
		Yes	31.7% (20)	40.2% (76)	
Previous ipsilateral BPPV	Previous BPPV of the same semicircular canal was considered positive, provided that BPPV had been treated with a repositioning maneuver and loss of nystagmus had been verified at least 90 days before the start of the symptoms of the current vertigo attack	No	93.8% (60)	92.7% (179)	0.785
		Yes	6.3% (4)	7.3% (14)	
Sulpiride intake	Sulpiride intake in the 24 h prior to the medical consultation	No	90.6% (58)	85.1% (166)	0.264
		Yes	9.4% (6)	14.9% (29)	
Betahistine intake	Betahistine intake in the 24 h prior to the medical consultation	No	87.5% (56)	87.7% (171)	0.968
		Yes	12.5% (8)	12.3% (24)	
Lateralization	Left or right	Left	36.5% (23)	49.5% (96)	0.073
		Right	63.5% (40)	50.5% (98)	

*Ipsilateral inner ear characteristics*					
Head thrust test	Positive ipsilateral head thrust test	No	80.6% (50)	78.4% (152)	0.700
		Yes	19.4% (12)	21.6% (42)	
Inner ear disease	Ipsilateral defined Ménière's disease, vestibular neuritis, sudden hearing loss or vestibular migraine were considered positive	No	100% (64)	95.9% (186)	0.099
		Yes	0% (0)	4.1% (8)	

An Epley Maneuver (EM)^9^ of the affected side was performed on patients in both groups. Patient consent was required before the EM. Patients who did not consent were excluded from the study.

**Figure 1:**
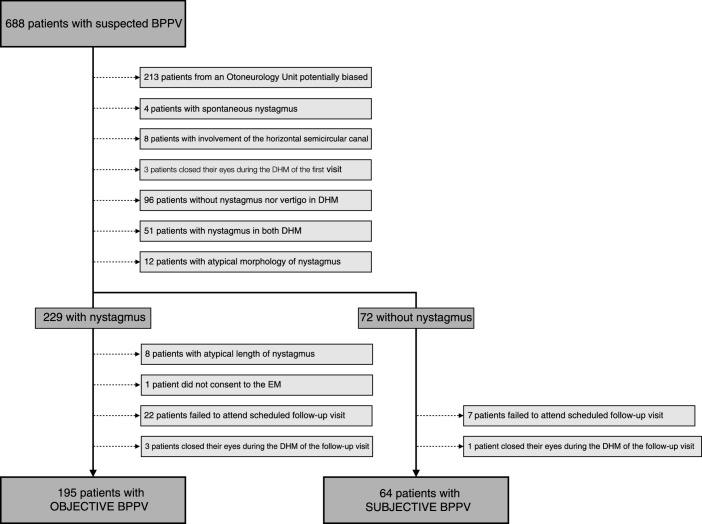
Screening of potential candidates for the study until the two groups for the study are formed.

Patients were given an appointment for a follow-up visit 7 days after the EM and those who failed to attend the follow-up visit or those who had closed their eyes during the DHM, thus making it impossible to determine the presence of nystagmus, were excluded from the study. In the follow-up visit, the DHM was repeated for the side that had been treated using the EM to check if nystagmus and vertigo was present in this position. Fig. 1 sketches the selection process.

The data obtained was analyzed in two different ways. Firstly, the success of the EM in the group with oBPPV was compared with its success in the group with sBPPV, both in terms of the resolution of nystagmus and also in terms of the resolution of vertigo in the DHM. Secondly, the data in Table 1 showing the percentages for each of the characteristics was compared to see if there were any differences between the two groups. Groups were compared using the *χ*^2^ test for nominal variables and the Mann–Whitney *U* test for osteoporosis (considered an ordinal variable). Osteoporosis was treated as an ordinal variable and, following the WHO guidelines, the categories “normal bone mass”, “osteopenia” and “osteoporosis” were used.^10^ In our analysis of this point, only those patients who had had a densitometry carried out up to 2 years before their hospital visit were taken into consideration. Migraine was treated as a dichotomous variable, although extra data on the evolution of migraine in each patient were obtained, emphasizing the presence of migraine headaches in the present or their disappearance before or after the last 2 years. Sulpiride and betahistine are drugs that are commonly used to treat BPPV in our country. Patients in our study were not prescribed these medications but some of them took them and because of that the intake of both drugs was analyzed.

In order to avoid biases for multiple comparisons, a Bonferroni correction was made, considering significant a *p*-value below 0.0026.

The protocol of this research study was accepted by the ethics committee of the main participating hospital (Id: CEIC-CHUIMI-2016/849).

## Results

During the time period of the study, a total of 688 patients suspected of having BPPV were recruited in the five Otoneurology Units. Initially, 213 patients were excluded because they came from centers where the presence of nystagmus was considered a criterion for referral and they could potentially bias the results obtained.

Next, 4 patients were excluded because they presented spontaneous nystagmus and 8 were excluded because the horizontal semicircular canal was affected. After carrying out the DHM on the left and right side, 3 patients were excluded because they closed their eyes during the DHM in the first visit; 96 patients were excluded because they did not present either vertigo or nystagmus during the maneuvers; 51 were excluded because they had nystagmus in the maneuvers on both sides; 12 were excluded due to the atypical morphology of their nystagmus.

Following on from this, the patients were separated into the oBPPV and sBPPV groups. After excluding the patients whose nystagmus lasted for 60 s or longer, those who did not consent to having the EM performed, those who failed to attend the scheduled follow-up visit and those who closed their eyes in the DHM during the follow-up visit, the sample size was 259 patients, of which 195 were in the oBPPV group and 64 were in the sBPPV group. The sBPPV group thus made up 23.8% of the sample.

The average age of the subjects was 59 years old and 72.2% of the subjects were female. Nystagmus was eliminated in 67.2% of the patients with oBPPV; 89.1% of the patients with sBPPV remained unaffected by nystagmus, thus showing a significant difference between the groups (*p* = 0.001). Therefore nystagmus appeared in 7 of the patients in the sBPPV group; only one of them commented that the nystagmus was not accompanied by vertigo. Vertigo in the DHM was eliminated in 50.8% of the patients with oBPPV and in 48.4% of the patients with sBPPV, thus there was no significant difference between the groups (*p* = 0.75).

Table 1 shows the prevalence of the different risk factors for the development of BPPV in the oBPPV and sBPPV groups. None of the studied variables reached significance, even before the Bonferroni correction. However, osteoporosis and migrane reached a *p*-value of 0.064 and 0.055 respectively. The variable osteoporosis was not significant in the Mann–Whitney *U* test; however, if the osteopenia and osteoporosis categories had been merged, the *χ*^2^ test *p*-value would have been 0.019. In relation to migraine, it was considered as being present when the patient mentioned a previous or current history of headaches with migraine-like characteristics, but a more exhaustive analysis of this variable revealed that the statistical significance was caused by patients who had suffered from migraines in the past before the current episode of vertigo.

In those patients who were taking vestibular suppressive medication, the percentage of sBPPV was not significant higher, although this fact was expected.

## Discussion

The results obtained from our sample support the hypothesis that, in most cases, sBPPV is a type of BPPV in which the EM should be attempted as a form of treatment. Although the definite diagnosis of BPPV requires observation of positional nystagmus,^1^ in some cases, otoconia particles free-floating in the canal may be minimal, but make the cupula deflect sufficiently enough to trigger symptoms of vertigo, but not the appearance of a nystagmus.^11^ Therefore, it is not necessary to identify a positional nystagmus to make the diagnosis of possible BPPV^1^ nor for the treatment of sBPPV.^11^

In our study, patients were examined without Frenzel glasses. Using the glasses during the DHM could have increased the number of patients in the oBPPV group at the expense of the sBPPV group, as this would have limited central suppression and thus nystagmus that was undetected by the naked eye would have been detected.^7^ One estimate is that this could occur in 10% of patients with sBPPV.^12^ However, using the glasses would have limited our ability to extrapolate from our results and compare them to most of the consulting rooms where BPPV patients are treated, as Frenzel glasses are not normally available there.

### Results of the EM in patients with sBPPV

The results of the EM in patients with sBPPV can be measured in two different ways. The first is by looking at the elimination of vertigo symptoms in the DHM. In this regard, the results of our group are similar to those described by most other authors, although they are lower than the results reported by Zhang et al.^13^ The second way is by measuring the percentage of patients who remain unaffected by nystagmus. By definition, nystagmus is not initially present in the sBPPV group. In the follow-up visit, 89.1% of the patients remained unaffected by nystagmus. This fact indicates two things. Firstly, the EM does not work in all cases of sBPPV. Secondly, the sensitivity of the DHM is not 100% when it is used to diagnose BPPV by detecting nystagmus and therefore it has to be emphasized that the classification in the sBPPV or oBBPV group is carried out attending to the exploratory findings during the first visit and not attending to the physiopathological reality of the inner ear.

Recently, it has been reported that the success of the EM depends on the intensity of the nystagmus observed during the DHM. A nystagmus of subjectively higher intensity correlates significantly with a worse prognosis for the EM,^14^ which could be related to the larger size of the otoliths.^15^ In this sense, sBPPV could be a type of BPPV caused by a small number of otoliths that are unable to cause nystagmus, as the results of the EM in the sBPPV group are better than in the oBPPV group.

### Factors related to the absence of nystagmus in BPPV

Although similar epidemiological and clinical features have been reported for patients with oBPPV and sBPPV,^16^ the incidence of the risk factors had not previously been reported. In this way, the present study contributes to the scientific literature by showing that none of the studied variables are more frequent in patients with sBPPV than in patients with oBPPV. Despite this fact, osteoporosis and migraine obtained *p*-values that would have been close to the significance in a monovariant analysis.

Although finally not significant, osteoporosis seemed more common in the group with sBPPV. If osteopenia and osteoporosis categories would have been merged, their *p*-value would have become 0.019, showing an association between sBPPV and Ca^2+^ disturbances. In the inner ear of the rat, the morphology of the otoconia changes when the rat suffers from osteoporosis, with the otoconia becoming larger in size and lower in density.^17^ These changes to the otoconia of patients with osteoporosis could cause nystagmus less likely to occur, like a biomechanical model^18^ has described, and thus explaining our results. In humans, osteoporosis could disturb local Ca^2+^ homeostasis in the inner ear,^19^ meaning that patients suffering from this disease are at higher risk of both BPPV onset and relapse.^20^ In our sample, within the group with sBPPV, no significant differences were found in the percentages of resolution of vertigo symptoms (*p* = 0.316).

We could not prove that migraine was more prevalent in the group with sBPPV; in our study the obtained *p*-value was 0.055. Patients with migraine have motion sickness susceptibility^21^ and this could lead one to think that some of the subjects with sBPPV experienced symptoms during the DHM due to motion sickness rather than due to the low otolith load in the posterior semicircular canal. However, this theory was rejected because the factor that was associated with sBPPV was the previous history of migraine rather than a current episode of migraine during the DHM. Patients with migraine have a higher risk of suffering from BPPV than the general population.^22^ The link between migraine and BPPV is unknown at present and could be related to vasospastic phenomena or to changes to the calcium channels.^23^ These phenomena could cause a change in the metabolism of the otoconia or in the ion concentration of the endolymph that might cause a change in the morphology of the loose otoconia in patients with migraine, thus making it more likely that their BPPV would manifest as sBPPV.

Finally, in our sample, no significant differences in the percentage of patients who had taken sulpiride or betahistine were found between the two groups. A later analysis also failed to find significant differences in the use of these medicines between these groups and the patients who had been excluded because of spontaneous resolution, and therefore our data indicates that the use of these two vestibular suppressants does not affect the results of the DHM for the placement of the patient in a diagnostic category. Therefore, it might not be strictly necessary to recommend that patients avoid taking vestibular suppressants before undergoing the DHM as it does not seem that the use of these medicines obscures the findings of the maneuvers.^24^

## Conclusion

The EM should be attempted as a form of treatment in sBPPV. This type of BPPV could be linked to a change in the morphology of the otoconia. Osteoporosis and migraine were not associated with a greater presence of sBPPV. The use of vestibular suppressants does not seem to affect the detection of nystagmus.

## Funding

This research received no specific grant from any funding agency in the public, commercial, or not-for-profit sectors.

## Conflicts of interest

The authors declare no conflicts of interest.
